# Comparison of Learning Effects of Virtual Reality Simulation on Nursing Students Caring for Children with Asthma

**DOI:** 10.3390/ijerph17228417

**Published:** 2020-11-13

**Authors:** Kyung-Ah Kang, Shin-Jeong Kim, Myung-Nam Lee, Mikang Kim, Sunghee Kim

**Affiliations:** 1 College of Nursing, Sahmyook University, Seoul 01795, Korea; kangka@syu.ac.kr; 2 School of Nursing, Hallym University, Gangwon-do 25949, Korea; ksj@hallym.ac.kr; 3 Department of Nursing, College of Health Science, Kangwon National University Samcheok, Gangwon-do 25913, Korea; myungnam81@gmail.com; 4 Laerdal Medical, Seoul 06725, Korea; Lena.Kim@laerdal.com; 5 Red Cross College of Nursing, Chung-Ang University, Seoul 06974, Korea

**Keywords:** asthma, care, children, high-fidelity simulation, virtual reality simulation

## Abstract

With the global pandemic of the coronavirus disease, virtual reality simulation (vSim) has emerged as a simulation educational method. The purpose of this study is to examine the learning effects of vSim by comparing three different educational modalities of nursing care for children with asthma. A quasi-experimental design with three different teaching methods, vSim, high-fidelity simulation (HFS), and vSim with HFS, were used in the study. The group using vSim with HFS showed the highest scores in knowledge, confidence in practice, and performance compared to groups using vSim or HFS alone. Simulation practice using vSim combined with HFS could be an effective educational method for nursing students.

## 1. Introduction

The emergence of the coronavirus disease 2019 (COVID-19) at the beginning of 2020 and the declaration of a pandemic in March 2020 led to the shutdown of activities, including education [1]. In the curriculum for undergraduate nursing students, clinical practice is considered one of the most important areas that must be experienced [2]. The unexpected crisis has led to the closure of colleges and universities and has disrupted academic activities, leading to a change to remote classes as an educational method, including clinical education in nursing schools. Therefore, nursing students are in the most troublesome of hospital-based educational settings [3].

In the current situation, where remote clinical educational training is required to protect students from COVID-19, a virtual reality simulation (vSim)-based educational method has been introduced to replace hospital-based clinical education in nursing schools in Korea. Hence, it is necessary to examine the effects of vSim to understand the implications for nursing and potential challenges for future clinical education.

The continued expansion in nursing education, particularly in nursing practice, has encouraged a wide variety of educational methods, such as simulation. Simulation has been applied widely in nursing education and has proven to be an effective and safe teaching alternative that provides opportunities to practice within a limited environment [4]. Further, with the rapid spread of technology, numerous innovative learning methods have become available. In simulation education, various simulation modalities, such as web-based simulation [5], multi-mode simulation [6], and vSim, are provided.

Types of vSim that use avatars have recently attracted attention by dynamic learning environments that reflect real-world situations [7]. They further help to accomplish the learning goal by presenting the patients’ clinical situation and enable repetitive learning [7]. VSim also has flexibility and high accessibility that enables long-distance learning anywhere, anytime [8]. More effective learning outcomes are expected to be seen by practicing the application and integration of knowledge, supplementing the limited simulation training time, and bridging the gap between theory and practice in nursing education [9]. With these benefits, education using vSim is regarded as a promising teaching method and began to be actively attempted in Korea [10].

The prevalence of asthma in Korean children has been reported to be 5–9%, and this rate has been on the rise due to recent environmental air pollution [11]. Asthma is a chronic inflammatory disease of the airways characterized by bronchial hyperreactivity and variable airway obstruction, which results in recurrent episodes of wheezing, breathlessness, chest tightness, and coughing that can vary over time and in intensity [11]. Asthma is characterized not only as an acute but also a long-term disease that requires accurate treatment and continuous management, and if not properly controlled, it may lead to hospitalization. Therefore, early awareness and coping ability are considered important. For nursing students, vSim and high-fidelity simulation (HFS) may enhance the nursing practice of caring for children with asthma through repetitive learning.

Many studies have emphasized the positive impacts of HFS by indicating improved variable outcomes of simulation education. On the contrary, however, some results have reported clinical performance worsened after HFS, or confidence had decreased in a situation where the patient’s condition worsened [12]. Owing to the differences in the effectiveness of simulation education in the reported studies [13,14], limited research is available to compare the effects of simulation educational modalities with vSim [5]. Identifying an effective way to prepare novice practitioners, such as nursing students, is a key concern for nurse educators, for whom robust evidence to support the effectiveness of different simulation teaching methods is required. Therefore, it is necessary to identify the most effective simulation education method by comparing the use of different simulation modalities.

The purpose of this study is to examine the learning effects of vSim by comparing three different educational modalities on nursing care for children with asthma. Our research seeks to determine the differences in vSim learning effects in (a) knowledge, (b) confidence in practice, and (c) performance among three groups of nursing students before and after the intervention.

## 2. Materials and Methods

### 2.1. Design

**Figure 1:**
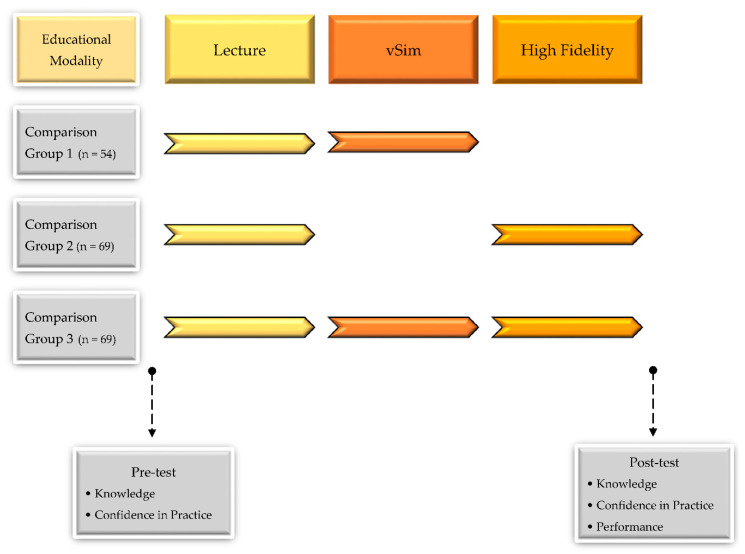
Research design.

This is a pre- and post-quasi-experimental study with three comparison groups. Three educational modalities were applied after a lecture: virtual reality simulation (vSim) for comparison group 1 (Com 1), HFS for comparison group 2 (Com 2), and a combination of learning methods (vSim with HFS) for comparison group 3 (Com 3) (Figure 1).

### 2.2. Setting and Participants

This study was conducted at three nursing schools located in different cities in South Korea. The schools in the three groups had similar numbers of nursing students and overall school capacity. The inclusion criteria applied to ensure homogeneity were as follows: (i) junior nursing students who have completed the pre-nursing course, fundamental nursing, and child development nursing with the equivalent of two credits using the same textbook; (ii) students with no clinical practice experience in the pediatric ward; (iii) students with no prior participation in either vSim or HFS classes. Of the 211 participants (Com 1: 60, Com 2: 76, Com 3: 75), 19 were excluded owing to incomplete responses. Finally, data of 192 participants (91.0%) were analyzed. The sample size was calculated using G*Power 3.1 based on one-way ANOVA as a major analysis method, a medium effect size of 0.25, 80% power, a significance level of 0.5, and three groups [15]. The desirable sample size was 159. Therefore, the number of participants in this study was considered acceptable.

### 2.3. Educational Interventions

**Figure 2:**
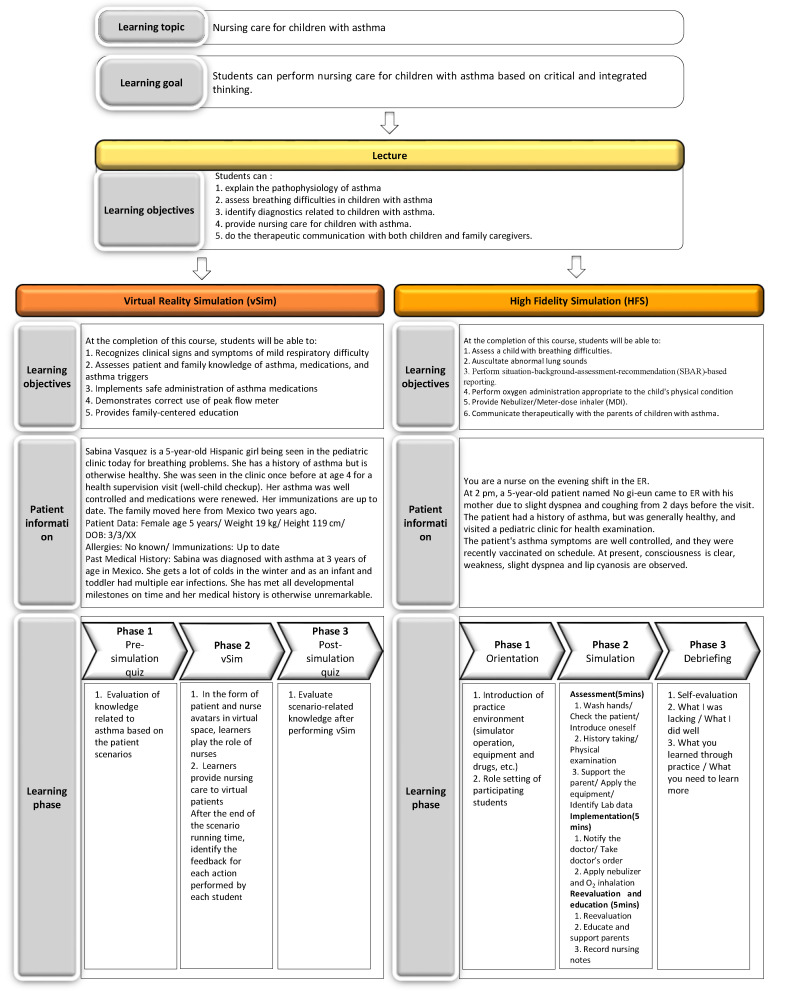
The flow of educational intervention.

In this study, three educational modalities (lecture, vSim, HFS) were applied on the participants who were caring for children with asthma. The goal of this education was to improve nursing students’ caring competency and the critical and integrated thinking for children with asthma. Based on the learning objectives established by the Korean Academy of Child Health Nursing [16] and the clinical nursing protocol [17,18,19,20] for children with asthma, the learning objectives connected to goals were set based on each of the three educational modalities. The learning objectives of the three educational modalities (lecture, vSim, and HFS) are linked. Based on the learning objectives of “lecture,” learning objectives of both vSim and HFS were reconfigured according to the strength of both vSim and HFS for more effective practice (Figure 2).

#### 2.3.1. Lecture

Educational content in the lecture focused on assessing pathophysiological mechanisms, related diagnostics, nursing care, and education for both patients and family caregivers.

#### 2.3.2. Virtual Reality Simulation (vSim)

A vSim scenario of a child with mild intermittent asthma (Laerdal Medical) was selected, and the scenario information was translated into Korean. The patient in the presented scenario was a 5-year-old girl who experienced asthma symptoms and was admitted to the emergency room. The educational content of the vSim consisted of respiratory assessment of the child with mild intermittent asthma who visited the emergency room, identification of mild respiratory difficulties, administration of drugs according to the doctor’s orders, and education regarding asthma prevention and treatment.

#### 2.3.3. High-Fidelity Simulation (HFS)

In HFS (SimJunior, Laerdal Medical) education, a scenario suitable for Korean nursing situations was developed based on the case of vSim patients. The objectives of the HFS education mode were to improve students’ competency in relation to the assessment of dyspnea with auscultation of lung sound in children with mild intermittent asthma, by reporting situation-background-assessment-recommendation (SBAR), administration of oxygen and drugs, and performance of therapeutic communication with parents. Furthermore, feedback through group reflection was provided in debriefing after the end of the practice.

### 2.4. Instruments

#### 2.4.1. Knowledge

The knowledge scale of nursing care for children with asthma was developed considering learning objectives by the researchers, who are also professors of pediatric nursing. It consisted of 16 questions in the areas of assessment (seven items) and intervention (implementation, four items; education, five items). Each item was scored as 0 (incorrect) or 1 (correct), and total scores ranged from 0 to 16. Higher scores indicate a higher level of knowledge. In the case of knowledge instruments, the researchers believe that Cronbach’s alpha is not appropriate with dichotomous scales that are coded as ‘right’ and ‘wrong.’ Subcategories of knowledge measurement items were organized based on learning objectives, and specific details of subcategories were presented in Supplementary Table S1.

#### 2.4.2. Confidence in Practice (CP)

In order to measure CP, the confidence tool for clinical performance developed by Kim [21] was modified and supplemented by focusing on the subject of this study: treating children with asthma. In other words, the CP scale, like performance, was developed by researchers to measure the sense of being able to perform with confidence, based on the contents of simulation applied in this study. It consisted of 10 items distributed into four categories: assessment (four items), planning (one item), intervention (implementation, two items; education, two items), and evaluation (one item), and it is scored on a 4-point Likert scale. The higher the score, the higher the confidence in caring for children with asthma. The Cronbach’s alpha of the scale was 0.932 (Supplementary Table S1).

#### 2.4.3. Performance

In order to measure performance, the researchers developed a performance tool based on the clinical performance tool developed by Lee [22] and taking into consideration the nursing process. In addition, based on the contents of asthma simulation education, this tool was developed to reference the learning objectives as defined by the researchers. It consisted of 11 items categorized into two groups: assessment (three items) and intervention (implementation, four items; education, four items). The higher the score, the better the student’s ability to care for children with asthma. Each item was scored as 0 (no) or 1 (yes), and total scores ranged from 0 to 11. The internal consistency reliability by KR-20 was 0.607 (Supplementary Table S1).

#### 2.4.4. Content Validity

Content validity for the tools was evaluated by four experts with backgrounds in research and nursing care who have worked for over 10 years in the pediatric ward at general hospitals. The content validity indices of each item that were above 80% fit the acceptance criteria.

### 2.5. Procedures

Data collection and clinical education were conducted from March to July 2020. During this period, owing to COVID-19, face-to-face lectures were conducted in small groups of less than 25 students. Prior to the lectures and practice, students agreed and signed up for small face-to-face lectures. All participants and researchers followed the guidelines provided by Korea Disease Control and Prevention Agency (KDCA) [23] for COVID-19 infection control (checking temperature, washing hands with soap, wearing masks, social distancing, etc.). During the study period, there were no participants infected with COVID-19.

In all three groups, the pre-test was conducted before the lecture, and subsequently, the lecture about nursing care for children with asthma was conducted. A professor lectured on the treatment of children with asthma for approximately 40 min. The researchers, who are professors from each of the three nursing schools, were the authors of the same textbooks on child health nursing. Before the lecture, they had three in-depth discussions to confirm the homogeneity of the lecture contents and methods.

After the lecture was completed, an orientation regarding vSim was conducted through self-learning, and a vSim ID was issued for each student. In this study, three steps comprising a pre-simulation quiz, vSim, and post-simulation quiz were applied. Suggested reading, documentation assignments, and guided reflection questions were used as references for self-evaluation and reflection. The vSim took approximately four days. After the vSim was completed, the Com 1 group performed the post-test. In the Com 3 group, HFS practice was performed after the vSim was completed, and post-test was conducted after the HFS was completed. In the Com 2 group, lecture, HFS, and post-test were conducted, respectively.

HFS education was conducted in three stages: orientation, simulation, and debriefing. It was composed of 3~5 students per group, and students played the roles of family caregivers. The running time for the simulation was 15 min, followed by a debriefing that lasted approximately 20 min. A performance evaluation and reflection time were then completed with group members.

### 2.6. Ethical Considerations

The study plan was reviewed and approved by the participating institutions’ research ethics boards (SIRB-2020010HR). After researchers explained the purpose, education content, and procedure, students willing to participate were recruited. It was explained that they could choose not to participate in the research at any time during the research process, and that there were no disadvantages in doing so. A consent form was provided prior to data collection and was voluntarily signed.

### 2.7. Data Analysis

The data were analyzed using IBM SPSS, version 25.0 (IBM, Armonk, NY, USA). Descriptive statistics were used to calculate frequencies and means for participants’ demographics and the degree of outcome variables in three groups. A paired t-test was used to measure the change within groups, and one-way ANOVA with post-hoc test (Duncan) was applied to compare the differences among groups and the homogeneity of pre-test among three groups.

## 3. Results

### 3.1. Homogeneity Test of Baseline Variables among Three Groups

Descriptive results and baseline equivalence among three groups with 192 participants revealed that 154 (80.2%) were female, and their age ranged between 20 and 30 (22.34 ± 1.88) years. No significant differences were observed in the baseline for knowledge among the three groups (F = 0.905, *p* = 0.406). However, there was a significant difference in the baseline in the CP (F = 107.930, *p* < 0.001) (Table 1).

### 3.2. Comparison of Mean Difference in Knowledge, Confidence in Practice, and Performance

#### 3.2.1. Knowledge

The mean differences of knowledge scores among the three groups are presented in Table 2. There were statistically significant mean differences (post-pre score) in knowledge among three groups (F = 3.709, *p* = 0.026). In the post-hoc analysis, the mean differences in both Com 1 and Com 3 were seen to be higher than in Com 2.

#### 3.2.2. Confidence in Practice

The mean differences in the CP scores among the three groups are presented in Table 2. There were statistically significant mean differences in CP among the three groups (F = 69.164, *p* < 0.001). Com 2 obtained a lower mean score than Com 1 and Com 3. Further, in the post-hoc analysis as well, mean differences in both Com 1 and Com 3 were seen to be higher than in Com 2.

#### 3.2.3. Performance

In the degree of total performance scores, a significant difference was seen among the three groups (F = 12.002, *p* < 0.001). In the post-hoc analysis, both Com 2 and Com 3 showed higher mean scores than Com 1. In categories of performance, significant differences were seen in assessment (F = 15.349, *p* <0.001) and education (F = 26.966, *p* <0.001). In the post-hoc analysis, Com 2 showed higher mean scores than both Com 1 and Com 3 (Table 3).

## 4. Discussion

As the COVID-19 pandemic provokes inevitable changes in nursing practice education, the search for alternative practical education that can effectively and safely cope with the changes is urgently required [24]. Until now, simulation-based education to improve integrated thinking and performance for clinical adaptation has become commonplace within undergraduate nursing curricula [25]. In this study, the learning effects of the simulation educational modality in nursing care for children with asthma were compared in knowledge, CP, and performance.

Compared to Com 1 (using vSim alone) and Com 2 (using HFS alone), Com 3 (using vSim with HFS) showed higher knowledge and CP. Regarding knowledge, the two groups (Com 1, Com 3) that applied vSim showed higher knowledge than the third group (Com 2). This positive effect can be seen as the result of systematic integration of knowledge through pre- and post-quiz in the vSim process. This is similar to results indicating that knowledge and CP improved after applying vSim to nursing students caring for patients with acute heart disease [9]. However, studies using HFS [26,27] did not show significant improvement in learners’ knowledge. To compensate for this weakness, vSim education that enables repeated learning can be complementarily used.

In CP, it can be assumed that the reason why the Com 2 group showed significantly lower CP is due to exposure to HFS education without repetitive self-study opportunities, after acquiring theoretical knowledge through lectures. Therefore, it is necessary to merge the simulation educational modality with the HFS modality to be effective. A study comparing HFS and blended simulation modality reported a significant improvement in nursing performance and confidence after using the blended simulation [9]. This is thought to have been a result of the positive effect of visual experience of real-life situations using vSim. VSim is constructed similarly to real-world situations, and it is possible to select and perform a nursing skill appropriate to the situation without being bound by skill proficiency. Furthermore, it provides the learner with an opportunity to actively learn without fear, which thereby may lead to an increase in the learner’s confidence.

In this study, the total performance score among the three groups was significantly higher in the Com 2 and Com 3 groups rather than the Com 1 group (vSim alone). This result implies it is necessary to provide an opportunity to apply knowledge learned during the lecture. A previous study reported that HFS education for children with asthma had desirable effects to improve clinical performance [28]. However, other studies reported web-based simulation programs have a significant learning impact on fundamental nursing, although they do not provide tactile practice [29,30]. This is also in accordance with the result that advanced cardiac life support (ACLS) education with vSim for clinicians and pharmacy students improved their ACLS performance more than traditional education [7,31]. These contradictory results suggest that hands-on practice has the most potential to improve the nursing student’s performance, and further studies need to confirm the effect of vSim on the performance level.

Based on this current study, vSim was identified as the effective alternative teaching modality in HFS education. Therefore, it is necessary to provide an opportunity to apply knowledge with indirect experience, such as vSim modality. As shown in this study, a vSim that allows self-practice before HFS education, rather than applying HFS education alone, is required to enhance the educational effects and improve clinical performance. The results of this study may be considered as meaningful evidence regarding learning effects of vSim that allow repetitive self-practice.

### 4.1. Clinical Implications

In this study, when exposed to HFS after repetitive self-practice by vSim, learning effects increased when compared to other methods. This indicates that various simulation educational modalities are needed to improve the HFS learning effect. Findings of the current study can serve as meaningful data to verify the usefulness of vSim in simulation education. The vSim, along with HFS simulation, can be a standard model in future nursing education. Further studies that may develop and apply various scenarios for vSim considering different situations in clinical pediatric nursing practice are necessary.

### 4.2. Limitations

The limitations of this study should be acknowledged. First, we used convenience sampling instead of a randomized controlled design. Second, there was no follow-up to determine the continuity effects of simulation educational modality. It would be beneficial to consider the longer-term effects of vSim, and a follow-up study is recommended.

## 5. Conclusions

To identify evidence of the educational effects using simulation, it is necessary to apply various educational simulation modalities. In nursing practice education, comparison and analysis of new methods that are rarely used are required. In the current study, vSim stands out as an effective simulation educational method. Simulation practice using vSim combined with HFS could be an effective educational method for nursing students. The study can be considered as evidence for the need for an educational method, such as vSim, that allows for repetitive self-practice before HFS, rather than applying HFS alone, to enhance educational effects and improve clinical performance.

## Figures and Tables

**Table 1:** Homogeneity test on baseline measures among three groups (N = 192).

Variables	Com 1 (n = 54)	Com 2 (n = 69)	Com 3 (n = 69)	F/χ^2^	*p*
Knowledge (range 0~16)	13.11 (1.34)	13.28 (1.20)	12.99 (1.28)	0.905	0.406
Confidence in practice (4-point Likert scale)	2.35 (0.50)	3.09 (0.27)	2.38 (0.39)	107.930	<0.001

Com (comparison group) 1: lecture and vSim; Com (comparison group) 2: lecture and high-fidelity simulation; Com (comparison group) 3: lecture, vSim and high-fidelity simulation.

**Table 2:** Mean comparison of knowledge, confidence in practice among three groups (N = 192).

Variables	Groups	Pre-Test	Post-Test	*t*	*p*	Mean Difference (Post-Pre)	F	*p*
		Mean (SD)	Mean (SD)			Mean (SD)		
Knowledge	Com 1^a^	13.11 (1.34)	13.81 (1.05)	−3.898	<0.001	0.70 (1.33)	3.709	0.026 * (a > b, c > b)
	Com 2^b^	13.28 (1.20)	13.49 (.93)	−1.668	0.100	0.22 (1.08)		
	Com 3^c^	12.99 (1.28)	13.74 (1.05)	−4.584	<0.001	0.75 (1.28)		
Confidence in practice	Com 1^a^	2.35 (0.50)	3.13 (0.32)	−10.220	<0.001	0.78 (0.56)	69.164	<0.001 * (a > b, c > b)
	Com 2^b^	3.09 (0.27)	3.20 (0.36)	−3.554	0.001	0.11 (0.25)		
	Com 3^c^	2.38 (0.39)	3.26 (0.40)	−17.794	<0.001	0.87 (0.41)		

Com (comparison group) 1^a^: lecture and vSim; Com (comparison group) 2^b^: lecture and high-fidelity simulation; Com (comparison group) 3^c^: lecture, vSim and high-fidelity simulation; SD: Standard deviation. *; post-hoc test (Duncan).

**Table 3:** Effects of performance among the three groups (N = 192).

Variables		Groups	Post-Test	F	*p*
			Mean (SD)		
Performance Total		Com 1^a^	0.78 (0.15)	12.002	<0.001 * (b > a, c > a)
		Com 2^b^	0.91 (0.14)		
		Com 3^c^	0.86 (0.15)		
	Assessment	Com 1^a^	0.70 (0.26)	15.349	<0.001 * (b > a, c > a)
		Com 2^b^	0.91 (0.20)		
		Com 3^c^	0.88 (0.20)		
	Implementation	Com 1	0.94 (0.11)	0.752	0.473
		Com 2	0.91 (0.14)		
		Com 3	0.93 (0.13)		
	Education	Com 1	0.69 (0.30)	12.966	<0.001 ^*^ (b > a, c > a)
		Com 2	0.92 (0.19)		
		Com 3	0.76 (0.28)		

Com (comparison group) 1^a^: lecture and vSim; Com (comparison group) 2^b^: lecture and high-fidelity simulation; Com (comparison group) 3^c^: lecture, vSim and high-fidelity simulation; SD: Standard deviation; *; post-hoc test (Duncan).

## Supplementary Materials

The following are available online at https://www.mdpi.com/1660-4601/17/22/8417/s1, Table S1: Knowledge; Table S2: Confidence in Practice (CP) and Table S3. Performance.
