# Supplementary material for: Comparison of Learning Effects of Virtual Reality Simulation on Nursing Students Caring for Children with Asthma
Source: Int J Environ Res Public Health. 2020 Nov 13;17(22):8417. doi: 10.3390/ijerph17228417 (PMC7696217; doi:10.3390/ijerph17228417)
Supplement: Supplementary file 1 [file ijerph-17-08417-s001.pdf]

Knowledge

NO.	Items	Right	Wrong
1	Exercise or pollen can cause asthma.	□	□
2	Asthma virus is present in the secretions of the respiratory tract and can be transmitted to the respiratory tract through coughing or sneezing, and is highly contagious.	□	□
3	Symptoms of asthma include wheezing, respiratory distress, and coughing attacks.	□	□
4	Children with severe asthma symptoms have severe cyanosis on physical examination, and hypoxemia and increased carbon dioxide pressure as a result of blood gas analysis.	□	□
5	Blood tests for asthma diagnosis include serum IgE, specific IgE, and eosinophil.	□	□
6	Skin reaction tests and symptom induction tests are tests to identify allergens.	□	□
7	The first nursing diagnosis for children with acute asthma attacks is “bronchospasm due to anxiety.”	□	□
8	If asthma persists, symptom control agents such as inhaled steroids are prescribed.	□	□
9	Corticosteroids improve air obstruction and reduce bronchial hypersensitivity.	□	□
10	Nebulizers are effective in depositing drugs directly into the airways.	□	□
11	Thoracic physiotherapy is a basic way to treat asthma.	□	□
12	The metered dose inhaler (MDI) delivers the drug directly to the airways, allowing the drug to spread to the narrowed airways.	□	□
13	Short-acting β_2-agonist is effective in relaxing bronchial muscles.	□	□
14	Cromolyn sodium can control asthma symptoms by relieving bronchospasm.	□	□
15	The primary prevention of asthma is to identify allergens and avoid them.	□	□
16	Asthma can be exacerbated by cigarette smoke, so smoking cessation is necessary at home.	□	□

Confidence in Practice (CP)

items	Not at all	Not Likely	Likely	Definitely
1. I can describe the goals of nursing a child with asthma.				
2. I can perform health assessments necessary for children with asthma.				
3. I can perform a nursing diagnosis for a child with asthma.				
4. I can identify the symptoms and signs associated with asthma early.				
5. I can plan the priorities of care for asthma symptoms.				
6. I can explain the tests needed for children with asthma and the purpose of the tests.				
7. I can check the symptoms of acute respiratory distress with asthma.				
8. I can apply nursing interventions to relieve acute respiratory distress caused by asthma.				
9. I can educate children and their families with asthma to relieve symptoms.				
10. I can evaluate the outcome of the nursing intervention performed on a child with asthma.				

Performance

NO.	Items	Yes	No
Assessment	The initial symptoms of asthma were intercostal depression and rapid breathing.	□	□
	At physical examination, cyanosis and blood gas test results (hypoxemia, increase in carbon dioxide pressure) were checked.	□	□
	Blood tests checked serum IgE, specific IgE, and eosinophil test results.	□	□
Intervention (Perform)	Raised the child's upper body.	□	□
	When β_2-agonist or steroid was administered, respiratory patterns, pulse, respiration and blood pressure were measured.	□	□
	Oxygen was supplied using an oxygen tent, oxygen mask, and nasal cannula.	□	□
	Drug administration using nebulizer was checked.	□	□
Intervention (Education)	After using the peak flow meter, the expiratory volume records were checked.	□	□
	When using the peak flow meter, it was checked that training was conducted to breathe vigorously and rapidly.	□	□
	Before using the metered dose inhaler (MDI), training was conducted to open the lid after shaking the drug up and down.	□	□
	Parent education on nebulization therapy was checked.	□	□
